# Quantifying the value of viral genomics when inferring who infected whom in the 2014–16 Ebola virus outbreak in Guinea

**DOI:** 10.1093/ve/vead007

**Published:** 2023-03-07

**Authors:** Alexis Robert, Joseph Tsui Lok Hei, Conall H Watson, Pierre-Stéphane Gsell, Yper Hall, Andrew Rambaut, Ira M Longini, Keïta Sakoba, Adam J Kucharski, Alhassane Touré, Sévérine Danmadji Nadlaou, Mamadou Saidou Barry, Thierno Oumar Fofana, Ibrahima Lansana Kaba, Lansana Sylla, Mohamed Lamine Diaby, Ousmane Soumah, Abdourahime Diallo, Amadou Niare, Abdourahamane Diallo, Rosalind M Eggo, Miles W Caroll, Ana Maria Henao-Restrepo, W John Edmunds, Stéphane Hué

**Affiliations:** Department of Infectious Disease Epidemiology, London School of Hygiene and Tropical Medicine, Keppel Street, London WC1E 6HT, UK; Centre for Mathematical Modelling of Infectious Diseases, London School of Hygiene and Tropical Medicine, Keppel Street, London WC1E 6HT, UK; Department of Biology, University of Oxford, South Parks Road, Oxford OX1 3RB, UK; Centre for Mathematical Modelling of Infectious Diseases, London School of Hygiene and Tropical Medicine, Keppel Street, London WC1E 6HT, UK; Epidemic Diseases Research Group Oxford, University of Oxford, Old Road Campus, Roosevelt Drive, Oxford OX3 7LG, UK; World Health Organization, Avenue Appia 20, Geneva 1211, Switzerland; UK Health Security Agency, Manor Farm Rd, Porton Down, Salisbury SP4 0JG, UK; Institute of Evolutionary Biology, University of Edinburgh, Ashworth Laboratories, Charlotte Auerbach Road, Edinburgh EH9 3FL, UK; Department of Biostatistics, University of Florida, 2004 Mowry Road, 5th Floor CTRB, Gainesville, FL 32611-7450, USA; World Health Organization Ebola Vaccination Team, Sonfonia T.7, Conakry, Guinea; Department of Infectious Disease Epidemiology, London School of Hygiene and Tropical Medicine, Keppel Street, London WC1E 6HT, UK; Centre for Mathematical Modelling of Infectious Diseases, London School of Hygiene and Tropical Medicine, Keppel Street, London WC1E 6HT, UK; World Health Organization Ebola Vaccination Team, Sonfonia T.7, Conakry, Guinea; World Health Organization Ebola Vaccination Team, Sonfonia T.7, Conakry, Guinea; World Health Organization Ebola Vaccination Team, Sonfonia T.7, Conakry, Guinea; World Health Organization Ebola Vaccination Team, Sonfonia T.7, Conakry, Guinea; World Health Organization Ebola Vaccination Team, Sonfonia T.7, Conakry, Guinea; World Health Organization Ebola Vaccination Team, Sonfonia T.7, Conakry, Guinea; World Health Organization Ebola Vaccination Team, Sonfonia T.7, Conakry, Guinea; World Health Organization Ebola Vaccination Team, Sonfonia T.7, Conakry, Guinea; World Health Organization Ebola Vaccination Team, Sonfonia T.7, Conakry, Guinea; World Health Organization Ebola Vaccination Team, Sonfonia T.7, Conakry, Guinea; World Health Organization Ebola Vaccination Team, Sonfonia T.7, Conakry, Guinea; Department of Infectious Disease Epidemiology, London School of Hygiene and Tropical Medicine, Keppel Street, London WC1E 6HT, UK; Centre for Mathematical Modelling of Infectious Diseases, London School of Hygiene and Tropical Medicine, Keppel Street, London WC1E 6HT, UK; Wellcome Trust Centre for Human Genetics, University of Oxford, Roosevelt Dr, Headington, Oxford OX3 7BN, UK; World Health Organization, Avenue Appia 20, Geneva 1211, Switzerland; Department of Infectious Disease Epidemiology, London School of Hygiene and Tropical Medicine, Keppel Street, London WC1E 6HT, UK; Centre for Mathematical Modelling of Infectious Diseases, London School of Hygiene and Tropical Medicine, Keppel Street, London WC1E 6HT, UK; Department of Infectious Disease Epidemiology, London School of Hygiene and Tropical Medicine, Keppel Street, London WC1E 6HT, UK; Centre for Mathematical Modelling of Infectious Diseases, London School of Hygiene and Tropical Medicine, Keppel Street, London WC1E 6HT, UK

**Keywords:** ebola virus (EBV), viral transmission, molecular epidemiology, contact tracing

## Abstract

Transmission trees can be established through detailed contact histories, statistical or phylogenetic inference, or a combination of methods. Each approach has its limitations, and the extent to which they succeed in revealing a ‘true’ transmission history remains unclear. In this study, we compared the transmission trees obtained through contact tracing investigations and various inference methods to identify the contribution and value of each approach. We studied eighty-six sequenced cases reported in Guinea between March and November 2015. Contact tracing investigations classified these cases into eight independent transmission chains. We inferred the transmission history from the genetic sequences of the cases (phylogenetic approach), their onset date (epidemiological approach), and a combination of both (combined approach). The inferred transmission trees were then compared to those from the contact tracing investigations. Inference methods using individual data sources (i.e. the phylogenetic analysis and the epidemiological approach) were insufficiently informative to accurately reconstruct the transmission trees and the direction of transmission. The combined approach was able to identify a reduced pool of infectors for each case and highlight likely connections among chains classified as independent by the contact tracing investigations. Overall, the transmissions identified by the contact tracing investigations agreed with the evolutionary history of the viral genomes, even though some cases appeared to be misclassified. Therefore, collecting genetic sequences during outbreak is key to supplement the information contained in contact tracing investigations. Although none of the methods we used could identify one unique infector per case, the combined approach highlighted the added value of mixing epidemiological and genetic information to reconstruct who infected whom.

## Introduction

The reconstruction of transmission trees, which link infectors and infectees in an outbreak of infectious disease, is a bedrock on which many public health decisions are made. Indeed, it is necessary not only to identify populations at risk but also to understand patterns of pathogen spread to effectively prevent or control infectious disease outbreaks. The study of transmission linkage and source attribution (i.e. who infected whom) during an epidemic is essential, as it allows a better understanding of the transmission processes (Lloyd-Smith et al. 2005; Blumberg and Lloyd-Smith 2013; Blumberg et al. 2014; Taube, Miller, and Drake 2021), the identification and quantification of factors associated with transmissibility (Faye et al. 2015; Robert et al. 2019; Leclerc et al. 2020), the reconstruction of historical epidemiological events (Wallinga and Teunis 2004; Ypma et al. 2012; Jombart et al. 2014; Campbell et al. 2018a; Robert et al. 2020), as well as a precise assessment of intervention strategies aiming at reducing transmission (Ferguson, Donnelly, and Anderson 2001).

Transmission tree reconstruction broadly falls into four categories: (1) traditional epidemiological investigations such as contact tracing, (2) statistical inference based on epidemiological factors (e.g. the timing or the location of cases), (3) phylogenetic reconstruction from pathogen genomic sequences, and (4) combined/evidence synthesis methods, which pool two or more of the aforementioned approaches. Contact tracing involves the identification and diagnosis of people who may have come into contact with an infected person, based on epidemiological data directly collected from the interviewed individuals (e.g. age, gender, when and where contact with a confirmed case occurred, and time of symptom onset). It is a standard approach that has been routinely applied to recent epidemic situations, such as the 2003 severe acute respiratory syndrome coronavirus (SARS-CoV) epidemic (Chen et al. 2005), the 2014–16 Ebola virus (EBOV) outbreak in Western Africa (Saurabh and Prateek 2017; Swanson et al. 2018), or the newly emerged SARS-CoV-2 epidemic (Kucharski et al. 2020; Sun and Viboud 2020). Despite its proven effectiveness, contact tracing is sensitive to reporting, ascertainment, or social desirability biases (Greiner et al. 2015). It is difficult to implement when parts of the infections are driven by pre-symptomatic or asymptomatic cases, when delays from infection to diagnosis are large or in the context of retrospective tracing, where recall bias hampers precise data collection. Furthermore, contract tracing is expensive and time-consuming and its success depends on the experience and capacity of healthcare workers to gather social histories.

An alternative means of inferring transmission chains relies on probabilistic algorithms through which the most probable transmission pathway of an infectious agent among all possible cases is inferred (Wallinga and Teunis 2004; Cauchemez and Ferguson 2012). The framework developed by Hens *et al.*, for instance, exploits the time interval among symptoms onsets, where the probability that a case has been infected by another case is estimated given their respective dates of symptom onset and the duration of the infectious period of the studied pathogen (Hens et al. 2012). These methods are also sensitive to sampling biases and heavily depend on accurate estimates of infection times, which makes them more suitable to the study of acute infections than the chronic ones. However, if all cases are treated as part of the same homogeneous outbreak, these inference methods have the potential to erroneously link individuals presenting with symptoms at the same time and place. Finally, they do not take into account the evolutionary history of the infecting strain(s), making them suboptimal in the case of outbreaks or epidemics caused by the concomitant circulation of more than one pathogen strain or genotype.

Gene sequences from rapidly evolving infectious agents also provide detailed information for transmission tree reconstruction. Through the inference of phylogenetic trees, which represent the evolutionary relationships among sampled pathogens, we can identify individuals infected by isolates that are more closely related to each other than what would be expected by chance, thus inferring epidemiological linkages. Phylogenetic reconstruction is widely used in transmission studies, sometimes in conjunction with temporal and spatial information (Bezemer et al. 2015). However, a pathogen phylogeny does not do justice to the underlying complexity of a transmission chain. Branching events in a phylogeny do not necessarily correspond to transmission events, and several transmission scenarios can explain a given phylogenetic cluster or tree topology (Hall, Woolhouse, and Rambaut 2015; Kenah et al. 2016). Moreover, phylogenetic trees are undirected and the direction of transmission between two phylogenetically linked individuals is not inferable from conventional sequence data, unless information about levels of intra-host diversity is available (Maio, Wu, and Wilson 2016; Didelot et al. 2017; Wymant et al. 2018).

Most recently, a new generation of methods combining genetic and epidemiological evidence has flourished (Cottam et al. 2008; Jombart et al. 2014; Kenah et al. 2016; Campbell et al. 2018a). Ypma *et al.* constructed an inference scheme that uses spatial, temporal, and genetic data simultaneously, but assumed that these data are independent of each other (Ypma et al. 2012). Alternative mixed approaches reconstruct transmission chains by combining an explicit epidemiological model with a model of molecular evolution to determine a unique likelihood from which the most likely transmitter for each case is inferred without having to reconstruct a phylogenetic tree. Recent developments of these methods can include the incorporation of contact matrix into the calculation of the transmission likelihood function (Campbell et al. 2019, 2018a).

The feasibility of transmission chain reconstructions will partly depend on the epidemiological characteristics of a given pathogen, such as its incubation period, symptoms, generation time, or known routes of transmission, which will determine the capacity an investigator has to identify an infection and sample it. Genetic characteristics can also play an important role in this context, especially when pathogen genomic information is explicitly incorporated in the reconstruction.

It is generally perceived that the addition of genetic information to epidemiological data enhances the accuracy of transmission reconstruction (Campbell et al. 2018b), yet the net value of this integration remains unclear. Debates remain as to whether incorporating genetic data is useful and cost-effective. The relative merits of these approaches, their degree of consistency, and applicability in outbreak or clinical trial situations remain poorly characterised. Moreover, the net value of pathogen sequencing in these specific contexts is unclear. New technologies for real-time sequencing are appearing (e.g. MinION (Jain et al. 2015)), forcing a reassessment of the utility of sequence data generated from primary samples during an outbreak, for either rapid intervention or routine surveillance.

EBOV infections present characteristics that make transmission chain reconstruction conceivable in most situations. The virus is associated with observable symptoms, has a short generation time, and its primary route of transmission, i.e. physical exposure to infected body fluids, is reasonably traceable. Although alternative routes of infection exist, such as fomite or aerosol transmission, an overwhelming majority of EBOV disease (EVD) cases have resulted from physical contact with a symptomatic patient (32). Viral genome sequences can also be valuable in epidemiological investigations of EBOV outbreaks. The mutation rate of the virus permits mutations to accumulate in the time between sampling of two individuals in a given transmission pair (i.e. an infector and a secondary case) so that their position within the transmission tree becomes distinguishable by genetic means, despite limited background diversity at the population level. These epidemiological or genomic characteristics make EBOV an attractive model to assess the accuracy of transmission chain inference (Campbell et al. 2018b).

We explored the accuracy, sensitivity, and propensity to biases of a range of methods for transmission chain inference, including phylogenetic reconstruction, statistical inference, and a method combining the two. These approaches were tested against empirical contact tracing data collected in Guinea during the late stages of the 2014–16 EVD outbreak in West Africa, with the aim of describing the degree of agreement between observed and inferred transmission trees.

## Material and methods

### The 2014–16 Guinea EBOV outbreak

Between December 2013 and May 2016, West Africa experienced the largest and most fatal outbreak of EVD in history with over 28,000 reported cases and 11,000 reported deaths (World Health Organization 2016). Guinea, Sierra Leone, and Liberia were mostly affected by multiple and persistent long chains of transmission, with smaller and non-sustained transmission or isolated cases observed in other countries including Nigeria, Mali, as well as the UK and the USA, leading to the declaration of a public health emergency of international concern by the World Health Organization on 8 August 2014 ().

### Contact tracing

The data used in this study originated from several large datasets collected in Guinea during the 2013–16 West Africa Ebola epidemic, as part of a wider control effort, subsequently collated and linked as described in Robert et al. (2019). Contact tracing information was collected by the Ministère de la Santé et de l’Hygiène Publique of Guinea (Ministry of Health and Public Hygiene). The dataset consisted of 860 confirmed cases of EVD assembled in eighty-seven transmission chains and forty-two isolated cases. These chains were reconstructed by trained field teams who conducted contact tracing interviews with cases, where possible, and their contacts.

The most likely infector or infectors were assigned to each case as suggested by the investigation. In addition to relevant epidemiological information such as location, age, and date of symptom onset for each case, a subset of individuals in the dataset were further linked with a database of consensus EBOV genome sequences that were collected during the outbreak for the purpose of genomic surveillance. We considered that Ebola transmission mostly occurred via direct contact with body fluids from infected individuals, as was done for the contact tracing analysis during the outbreak. We therefore ignored alternative mechanisms of transmission with potentially different epidemiological characteristics (such as aerosols). This is in line with local investigations suggesting that the most pre-eminent routes of transmission during the 2014–16 EVD outbreak were nosocomial, funeral, and household transmission (Faye et al. 2015), with the latest being the main route in the late stages of the outbreak (Robert et al. 2019).

### Viral genomic data

A total of 398 EBOV full genome sequences (18,503 nt) from the 2014–16 Guinea outbreak were matched to the transmission chain database using national ID and sequence ID. We identified 178 cases from the transmission chains reconstructed by contact tracing investigations for which viral sequence data were available.

### Study cohort

**Figure 1.**
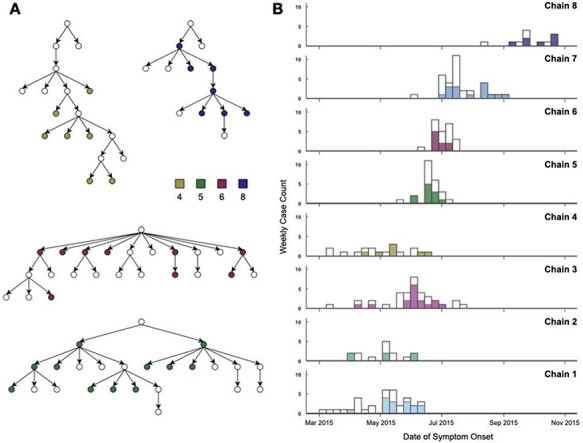
(A) Example of four transmission chains reconstructed from contact tracing investigations and included in the study. The chain identification number is indicated in the colour-coded legend. Each circle represents a case, and each case is connected to its assigned infector by an arrow, with the direction of the arrow indicating the direction of transmission. Filled circles correspond to cases linked to an available EBOV genome sequence. (B) Stacked bar chart depicting the weekly incidence of reported EVD cases in the eight transmission chains, from March to November 2015. Filled bars show weekly counts of reported EVD cases with available sequence data and are colour-coded according to their transmission chains in the contact tracing investigations. All the transmission chains are displayed in Supplementary Fig. S1.

Since we aimed to analyse the impact of sequencing on inference of transmission links, we restricted the study to the transmission chains identified by the contact tracing investigations with the highest proportion of cases that had been matched to a genome sequence (199 confirmed EVD cases in eight separate chains of transmission) (Supplementary Fig. S1). Of these 199 individuals, the study cohort consisted of the eighty-six cases (43.2 per cent) that were linked to their respective consensus EBOV genome sequence. In each chain, the proportion of cases linked to EBOV genomes ranged from 38 per cent (Chain 6) to 54 per cent (Chain 8; see Table 1). Date of symptom onset was recorded for all but nine cases (four cases in Chain 1, one in Chain 3, one in Chain 4, and one in Chain 8). We inferred six of them retrospectively from the date of confirmed death due to EVD, using the distribution between the date of symptom onset and the date of death in the dataset, and discarded the last three since the onset date could not be deduced from other data (two from Chain 1 and one from Chain 8). The selected chains are presented in Fig. 1 and Table 1.

**Table 1.** Characteristics of the selected EBOV chains of transmission.

Chain no.	No. of cases	No. of sequenced cases (%)	No. of generations	Duration (days)
1	37	15 (41)	10	97
2	15	6 (40)	6	66
3	35	16 (46)	10	130
4	18	7 (39)	8	68
5	24	11 (46)	5	50
6	24	9 (38)	4	34
7	33	15 (46)	6	86
8	13	7 (54)	6	71

### Phylogenetic reconstruction

We reconstructed the maximum-likelihood (ML) phylogeny of the selected eighty-six EBOV sequences and 1,446 publicly available EBOV full genomes (see Supplementary Table S1 for details). Sequences were aligned using the software mafft v7.453 (Katoh and Standley 2013) and reconstructed in IQ-TREE v1.6.12 (Nguyen et al. 2015), under the best-fitting model of nucleotide substitution (TIM + F + R2), as determined by the ModelFinder option implemented in IQ-TREE. The tree was rooted against a set of twelve EBOV full genomes sampled during the 1976 Zaire outbreak (GenBank accession numbers: KY425630, KY425647, MH121168, KY425649, KY425637, KY425656, MH121166, KC242791, KY425652, KM655246, KY425639, and KC242801). Branch supports were estimated by standard non-parametric bootstrap analysis with 1,000 replicates.

Phylogenetic clusters including EBOV sequences from the studied outbreak were extracted from the abovementioned tree and their phylogeny independently reconstructed as described earlier.

### Statistical inference of transmission pathways

We used the R package *outbreaker2* to infer the transmission trees connecting the eighty-six sequenced cases from (1) the onset dates alone (subsequently referred to as the epidemiological approach) and (2) the onset dates and the viral genomic sequences of the cases (subsequently referred to as the combined approach) (Campbell et al. 2018a:).

The model implemented in *outbreaker2* infers connections among cases, using a Metropolis–Hastings algorithm with Markov Chains Monte Carlo (MCMC) to sample from the posterior distribution of parameters and transmission trees. For each case $i$, the model estimates the infection date ${t_i}$, the infector ${\alpha _i}$, and the number of missing generations among them ${\kappa _i}$. The mutation rate of the virus $\mu $ and the proportion of sampled cases $\rho $ are also estimated during the inference procedure.

The input data needed to run the models include the distribution of the serial interval ($w$) and latent period ($f$) of the virus, along with the genetic sequence (${s_i}$) and the onset date (${T_i}$) of each case. The likelihood associated with each case $i$ is written as


$$ likelihood \, = \, & genome \ sequence \, \times \, serial \ interval \cr & \times \, latent \ period \, \times \, missing \ cases $$

(1) $${L_i}\, = \,{\Omega ^{{\kappa _i}}}({s_i}|\,{s_{{\alpha _i}}},\,\mu )\,\,x\,{w^{{\kappa _i}}}({t_i} - \,{t_{{\alpha _i}}}|{\kappa _i})\,x\,f\left( {{t_i} - {T_i}} \right)\,x\,p({\kappa _i}|\rho )$$

where ${\Omega ^{{\kappa _i}}}$ represents the genomic component of the likelihood, given the number of generations and the sequences of the connected cases. In the epidemiological approach, the transmission links were inferred without using the genomic component so that only the onset dates were informative of the connection among the cases, which correspond to setting ${\Omega ^{{\kappa _i}}}({s_i}|{\alpha _i},\,{s_{{\alpha _i}}},\,{\kappa _i},\,\mu )\, = 1$ for every case in Equation (1).

The distribution of the latent period was set using a Gamma distribution of mean 9.1 days and SD 7.3 days (WHO Ebola Response Team 2014), while the distribution of the serial interval was described by a Gamma distribution of mean 14.2 days and SD 7.1 days (Faye et al. 2015).

In *outbreaker2*, the number of importations in the dataset is estimated from the input parameter $\lambda $. This process is presented in detail in the study describing *outbreaker2*, but in short, the higher the $\lambda $ value is set, the higher the threshold value, and the lower the number of cases set as importations (i.e. the level of likelihood considered ‘plausible’ is less strict). In this study, we considered a high value of $\lambda \,$ for both scenarios ($\lambda = 3$), which reduces the number of connections deemed implausible. In the Supplementary Figs S4–S6, we present the results obtained with a lower value of $\lambda $ and show the limited impact of this parameter on our conclusions.

The MCMC chains were run for 20,000 iterations with a thinning frequency of one-fiftieth. A burn-in period of 2,000 iterations was chosen, leaving 360 sampled trees describing the posterior distribution. MCMC traces of the posterior distribution were used to assess mixing visually (Supplementary Fig. S2).

### Visualisation and comparison of methods

The inferred transmission trees were represented as a matrix in which each element corresponds to an infector–infectee pair. In this matrix, an element takes a value of 1 if its corresponding infector–infectee pair is present in the transmission tree and 0 otherwise. By representing each transmission tree in a posterior collection as a matrix, and computing the sum of these matrices, a matrix in which each element represents the posterior frequency of its corresponding infector–infectee pair, i.e. the number of times the transmission pair appears in the posterior collection, was obtained. These matrices were visualised as ‘alpha plots’, where the transmission trees reconstructed using the two inference methods tested (i.e. statistical inference and combined approach) were compared to those identified by contact tracing investigation. In these plots, the radius of each circle is proportional to the posterior frequency (Supplementary Fig. S3). Alpha plots were drawn using *outbreaker2*.

## Results

### Epidemic curves

The weekly incidence of the 199 EBOV cases in the eight studied chains of transmission was plotted over the time of the outbreak (Fig. 1 and Supplementary Fig. S1). All cases were drawn from the late stage of the epidemic with dates of symptom onset spanning over a period of 8 months from early March 2015 to November 2015. This period largely coincides with the Guinea EBOV vaccine trial (Henao-Restrepo et al. 2017), which would have been expected to limit the size of these transmission chains.

Six of the eight identified chains included symptom onset dates between March and August 2015. Of the 196 cases with recorded or inferred symptom onset date, 180 (91.8 per cent) started developing symptoms before August, with the earliest recorded symptom onset date being 7 March 2015 (Chain 1) and the latest being 23 October 2015 (Chain 8). Chain 8 was notably later, with cases recorded between August and November 2015. The mean duration (time difference between the earliest and latest date of symptom onset) of a chain of transmission was 79.1 days, with a range between 34 days (Chain 6) and 130 days (Chain 3).

### Phylogenetic inference

**Figure 2.**
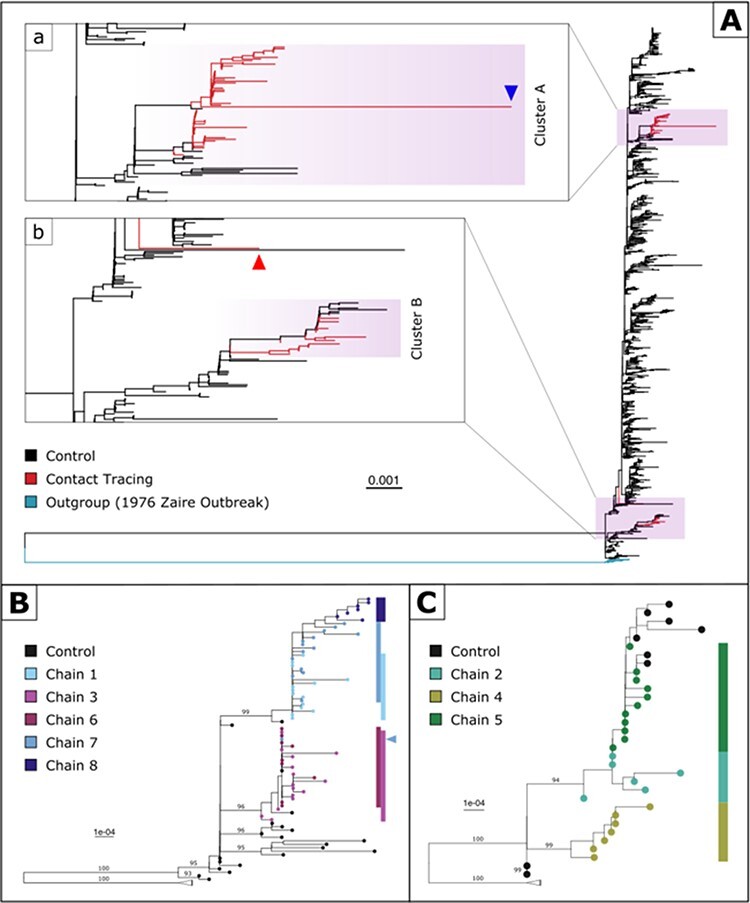
(A) Maximum-likelihood phylogeny of 1,532 full-genome EBOV sequences, with eighty-six from the studied population (red) and 1,446 as controls (black). The phylogeny is rooted against twelve sequences sampled during the 1976 Zaire Ebola outbreak (blue). Branch lengths represent nucleotide substitutions per site (see the scale bar). Close-up views of the two phylogenetic clusters are shown in subpanels (a) and (b). The blue arrow in subpanel (a) indicates the position of an abnormally long branch due to low sequencing quality (from Case Y in Chain 1). The red arrow in subpanel (b) indicates the position of an isolate (Case X in Chain 3) that is inferred to be phylogenetically distant from either cluster, thus being likely to have been misallocated by contact tracing. (B) Maximum-likelihood phylogeny of the eighty-one full-genome EBOV sequences in Cluster A. Branch supports were evaluated using standard non-parametric bootstrap with 1,000 replicates (only values above 90 per cent are shown). The coloured strips indicate the general positions of sequences from the five chains of transmission in the phylogenetic tree. The blue triangle indicates the position of the isolate from Case Z in Chain 7 inferred to be in the monophyletic clade containing mostly sequences from Chains 3 and 6. (C) Maximum-likelihood phylogeny of the thirty-three full-genome EBOV sequences in Cluster B. Branch supports were evaluated using standard non-parametric bootstrap with 1,000 replicates (only values above 90 per cent are shown).

The maximum-likelihood phylogeny of the studied sequences is shown in Fig. 2. Sequences from the eight transmission chains formed two distinct monophyletic clades (labelled Clusters A and B), suggesting multiple introductions of the circulating EBOV lineages in the studied area (Fig. 2A).

Overall, there was a good agreement between the phylogenetic reconstruction and contact tracing investigation: sequences from a given transmission chain clustered together in the ML trees (Fig. 2B and C), thus confirming the linkages established by contact tracing investigation, with three exceptions: (1) one sequence sampled from putative Chain 3 (subsequently referred to as Case X, indicated by a triangle in Fig. 2A, panel b) was genetically distinct from all others and clustered outside of Clusters A and B, suggesting misassignment during contact tracing investigations. (2) One sequence from Chain 7 (labelled Case Z, indicated by a triangle in Fig. 2B) clustered with sequences from Chains 3 and 6, suggesting an allocation to the wrong chain of transmission (Chain 7) as a result of a misrecorded symptom onset date. This was further supported by the observation that this patient’s timing of symptom onset was significantly later than most cases in Chains 3 and 6 but consistent with the range of symptom onset dates in Chain 7 to which this case was (erroneously) assigned by contact tracing investigations. (3) Finally, a sequence with an abnormally long branch length was identified (labelled Case Y, corresponding to the arrow in Fig. 2A, panel a). Further examination revealed that the sequence was of lower quality than the rest of the set (14.6 per cent of unidentified nucleotides, compared to an average of 5.4 per cent per sequence).

There was also further evidence of unobserved intermediate transmissions from the ML tree with sequences from Chains 3/6 and 1/7 appearing to be intermingled (see Fig. 2B) despite being assigned to separate chains of transmission by contact tracing. Moreover, ancestral relationships among independent chains were observed, with Chain 8 and Chain 5 being extensions of Chain 7 and Chain 2, respectively, albeit only weakly supported by bootstrap values. The presence of unobserved links between chains is also further supported by the minimal genetic differences observed in the sequence dataset. Pairwise nucleotide differences ranged from 0 to 10 nucleotide substitutions over 18,500 positions within a contact tracing chain and between 0 and 64 nucleotide substitutions across chains.

### Comparison between epidemiological and combined approaches

**Figure 3.**
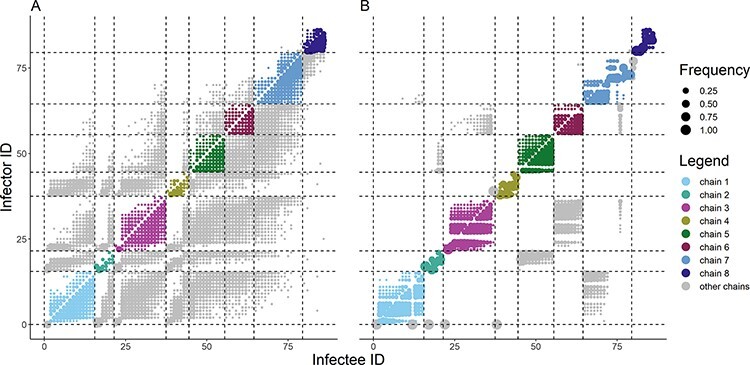
Alpha plots representing the posterior distribution of ancestry assignments obtained using the dates of symptom onset only (Panel A), and the dates of symptom onset and the genetic sequences (Panel B) as the data source. The radius of each circle indicates the frequency of a given transmission pair in the posterior distribution (i.e. posterior frequency). An imported case is assigned an infector of index 0. Circles representing within-chain transmission pairs are colour-coded by the chain of transmission. Circles representing cross-chain transmission pairs are coloured grey.

The transmission trees inferred by the epidemiological and the combined approach differed greatly. In the epidemiological approach, all cases were linked in the same transmission tree (i.e. there was one importation per inferred tree), although different cases were identified as the imported one across the sampled trees (three cases were classified as the importation in more than 20 per cent of the trees). Using only the onset date was not enough to identify a reduced pool of infectors for each case: on average, there were forty-four infectors per case, with a maximum of sixty-six infectors (Fig. 3A). On the other hand, there was less variance among the transmission trees inferred in the combined approach: there were five independent identified importations, with the same five cases being inferred as importations in more than 99 per cent of the sampled trees. The number of potential infectors per case was much lower than that in the epidemiological approach (eleven infectors on average, with a maximum of 20) (Fig. 3B). Adding the pairwise distance among genetic sequences to the inference framework was key to identifying independent transmission trees and reducing the number of plausible scenarios of transmission.

In the combined approach, only fifteen of the eighty-six individuals were assigned the same infector in more than 75 per cent of the trees (i.e. their most likely infector was clearly identified), whereas forty-four cases had more than five infectors assigned in at least 5 per cent of the sampled trees. This shows that in the majority of cases, the combined approach was not able to identify a pool of less than five potential infectors, indicating that combining genetic sequences and onset dates was not sufficient to reconstruct who infected whom.

### Comparison between inferred trees and contact tracing investigations

In the combined approach, the cases tended to group in agreement with the chains determined during the contact tracing investigation (Fig. 3). In the epidemiological approach, the chains were intermingled: the potential infectors of each case did not belong to the same contact tracing chain as their inferred recipients. Cases from Chain 8 were the only exception. They were mostly linked to other cases from Chain 8 or to the latest cases from Chain 7. This may be explained by the fact that most cases from Chain 8 were reported more than 2 months later than the other chains (Fig. 1).

**Figure 4.**
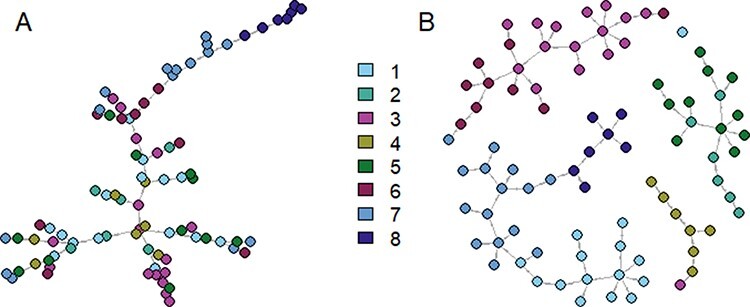
Consensus transmission trees inferred with each approach. Each circle represents a case, and each case is connected to its most likely infector in the posterior distribution of ancestry assignments (the direction of the arrow indicates the infector–infectee relationship). Circles are colour-coded according to their transmission chain in the contact tracing investigations (see Fig. 1). (A) Consensus transmission tree obtained with the epidemiological approach; (B) consensus transmission tree obtained with the combined approach.

We generated the consensus trees of the epidemiological and combined approach to highlight the trends represented in the alpha plots of Fig. 3. These trees were generated by linking each case to their most likely infector and colouring the cases by the contact tracing chain they belong to (Fig. 4). Given the number of infectors per case across the sampled trees, the consensus trees ignore a lot of information and are mostly useful to identify trends (especially in the epidemiological approach), while the alpha plots (Fig. 3) give a more complete description of the results generated by the inference. The consensus tree of the epidemiological approach consisted of a single network linking all cases of the contact tracing chains (Fig. 4A). Cases from a given chain were not necessarily linked to each other, but mostly intermingled along the network, with the exception of cases from Chain 8. These clustered together, as expected, and were linked to the latest cases from Chain 7. On the other hand, with the combined approach, cases were clustered in four distinct networks, within which more than one transmission chain was represented, alongside one isolated case (Fig. 4B). These four groups of transmission suggest that infections in some contact tracing chains may have been seeded by the same importation event:

1. Chains 1, 7, and 8 were grouped together, except for one case from Chain 7. The latter was grouped with cases from Chain 6 instead (identified as Case Z during the phylogenetic reconstruction) and one case from chain 1 (previously identified as Case Y).
2. Cases from Chains 2 and 5 were exclusively grouped together.
3. Cases from Chains 3 and 6 were grouped together, except for one case from Chain 3, which was grouped with cases from Chain 4 (previously identified as Case X).
4. All cases from Chain 4 were grouped together.

The clusters generated by the epidemiological and combined approaches were robust to changes in the importation threshold $\lambda $ (Supplementary Figs S4–S6): The number of importations increased abruptly in the epidemiological approach when $\lambda $ was reduced; however, the method was not able to identify which cases were imported (i.e. all cases are classified as importations in some of the iterations). This increase in the number of importations was likely due to the small variance between the likelihood of connections. On the other hand, the groups highlighted by the combined approach in Fig. 4 are still visible in Supplementary Fig. S5, showing the high likelihood of connections between chains in Groups 1, 2, and 3.

The alpha plots indicated that the misplaced cases were exclusively associated with cases from different transmission chains (Fig. 3). These three cases (i.e. the isolated importation from Chain 1 and the two misplaced cases) had already been identified in the phylogenetic analysis. Therefore, according to their genetic sequences, these infections were deemed unrelated to the transmission chain they were assigned to by contact tracing investigations.

**Figure 5.**
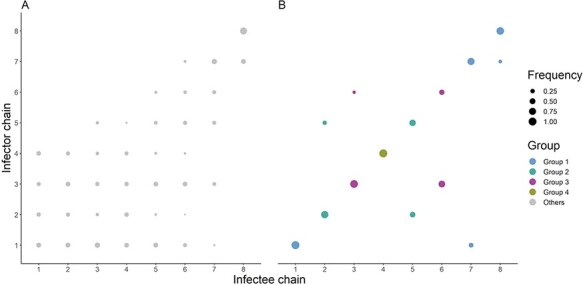
Alpha plots representing the connectivity between the contact tracing transmission chains in the epidemiological approach (Panel A) and the combined approach (Panel B). The three misplaced cases identified in Fig. 4 were removed. The radius of each circle corresponds to the frequency of transmission among chains, which was computed as the average proportion of infection of cases from the infectee chain by cases from the infector chain, across all iterations. Imported cases were not taken into account in the computation of the frequency. Circles representing within-group transmission pairs are colour-coded by group (as observed in Fig. 4).

We explored the links among the contact tracing chains within the four groups identified in the consensus trees. To do so, we excluded the three misplaced cases and calculated the proportion of connections between each chain: for each contact tracing Chain X, we computed the average proportion of the infectors of cases from Chain X who belong to Chain Y over the sampled trees in both approaches (Fig. 5). In the epidemiological approach, all chains were intermingled and no independent grouping could be identified. With the combined approach, we observed that all the cross-chain transmissions reported in the sampled trees followed the groups presented in Fig. 4. In Group 2, we were not able to highlight a direction of transmission between chains. Indeed, cases from Chain 2 were potential infectors of cases from Chain 5 and vice versa. We observed a similar pattern for Chains 3 and 6 in Group 3. In Group 1, there was a clear direction of transmission from Chain 1 to Chain 7 and from Chain 7 to Chain 8. There were only a small number of transmissions from cases belonging to Chain 7 to cases from Chain 1 (less than 5 per cent of the transmission links towards Chain 1). Finally, the cases from Chain 4 were not connected to any other chain.

**Figure 6.**
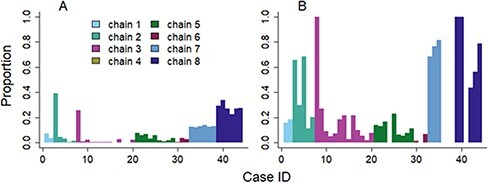
Proportion of sampled trees where the cases are linked to their contact tracing infector, in the epidemiological approach (Panel A) and the combined approach (Panel B).

In the contact tracing investigations, forty-four of the sequenced cases were linked (directly or through unsequenced cases) to another sequenced case. For each of these forty-four cases, we computed the proportion of iterations where they were linked to the same infector as in the contact tracing investigations (Fig. 6).

In the epidemiological approach (Panel A), no cases were linked to their contact tracing infector in more than 40 per cent of the sampled trees. Two cases belonging to Chains 2 and 3 were matched to their infector in more than 20 per cent of the trees, because their onset date was among the earliest of the study sample, and therefore they had a limited number of potential infectors. Similarly, cases from Chains 7 and 8 have higher proportions of matching infectors because they are temporally isolated from the other chains and have fewer potential infectors. Otherwise, the percentage of matching infectors was below 5 per cent.

In the combined approach (Panel B), we observed three groups of cases:

- Group 1 consisted of ten cases linked to their ‘contact tracing’ infector in at least 50 per cent of the sampled trees. The infector identified by contact tracing was also their most likely infector in the inferred transmission matrix, which indicates that the combined approach supported the conclusions from the contact tracing investigations.
- Group 2 consisted of fifteen cases linked to the contact tracing infector in 10 to 50 per cent of sampled trees. In this case, the ‘contact tracing’ infector was part of the pool of potential infectors but was not identified as the most likely. Either they belonged to a set of equally likely infectors, or there was another case that was more often classified as the infector.
- Group 3 consisted of twenty cases almost never linked to the contact tracing infector (the proportion of iterations was below 10 per cent). In this case, the combined approach was in clear disagreement with the contact tracing investigations. Although the cases belonged to the same transmission chain, they were unlikely to be linked, indicating that there were other cases belonging to this chain whose genetic sequences were more similar to that of the infectee.

## Discussion

We explored the ability of different data sources and inference methods to reconstruct the transmission trees during the late stages of the 2013–16 EVD outbreak in Guinea. Using a dataset of eighty-six sequenced cases, linked together in eight transmission chains by contact tracing investigations, we ran a set of phylogenetic and inference analyses using their genetic sequences and timing of infection. We found that, in this setting, individual data sources (i.e. either epidemiological or genomic) were insufficiently informative to accurately reconstruct the transmission trees. The phylogenetic analyses were globally consistent with the contact tracing investigations (i.e. most cases from each transmission chain were grouped in the same cluster) while disproving a number of links mistakenly inferred from contact tracing investigations. Furthermore, phylogenetic reconstruction confirmed the presence of multiple independent importations of the virus in the studied area, while also highlighting links among transmission chains deemed to be separate through contact tracing, likely because of unsampled links. However, direct linkage and direction of transmission could not be inferred from genome analysis alone.

The inference of transmission trees from the symptom onset dates of the cases proved suboptimal. This approach failed to distinguish different transmission chains, most likely due to the large amount of overlapping onset dates and infectiousness periods across the sampled individuals. The average number of potential infectors per case across the inferred trees exceeded forty, and all cases were grouped in a single, large transmission chain, in contradiction with the contact tracing investigations and phylogenetic reconstruction. This observation suggests that inference solely based on the timing of infections is insufficient to gain insight into the history and patterns of transmission when multiple transmission chains are ongoing simultaneously.

The combined approach, whereby both the onset date and genetic sequences are used to infer the transmission trees, was able to (1) identify a reduced pool of potential infectors for each case, (2) highlight which contact tracing links were inconsistent with the sequence data, and (3) identify strong links among certain chains. Therefore, combining the data sources was crucial to gain more insights into the dynamics of transmission.

These findings have multiple implications for data collection and outbreak control. First, the fact that the transmission dynamics captured by the contact tracing investigations was consistent with most of the sequence data confirms that timely contact tracing is an effective way to assess the scope of transmission during Ebola outbreaks. However, few discrepancies between the inferred transmission trees and the contact tracing chains suggest that there is potential for bias in contact tracing investigations. Reconstructing the exhaustive history of contacts for a given case can be challenging, mostly because of recall bias, logistical issues, or the identification and enrolment of the contact persons (Greiner et al. 2015; Swanson et al. 2018). Our study shows that sequencing of cases can help address this challenge, by identifying a reduced pool of potential infectors per case (and highlight connections missed by epidemiological investigations) and excluding unrelated cases where transmission links would be implausible given the differences in genetic sequences. Since only a proportion of the cases can be sequenced during EVD outbreaks, the dataset reconstructed by contact tracing investigations integrated a larger proportion of the total number of infected individuals and presented a more thorough description of the transmission dynamics during the outbreak. The overall agreement between the combined inference approach and the contact tracing investigations suggests that the latter enabled the gathering of an adequate core of information for the identification of most contacts. Therefore, in this case, the contact tracing dataset was a reliable data source to study outbreak dynamics and the factors associated with onward transmissions. This may not be the case in all outbreak investigations, especially when asymptomatic individuals, which are more challenging to identify, are involved.

The integration of genetic sequences in the inference method also permitted the identification of links among transmission chains, which had been deemed unrelated from the contact tracing investigations. Indeed, certain transmission chains appeared intermingled (i.e. the inferred trees showed several cross-chain transmissions, in both directions), whereas others branched off an earlier chain (e.g. certain cases from Chain 7 were linked to those from Chain 1). These links were not recovered by contact tracing investigations, effectively resulting in an underestimation of the length and scope of the transmission chains and indicating that the number of concurrent chains stemming from independent importations was lower than expected. We observed that the transmissions among chains classified as independent by the contact tracing investigations remained when we allowed a larger number of independent importations in the inference of the transmission chains, which shows that the inference method considers them at least as likely as most intra-chain links. Again, this indicates that viral genome comparison conclusively supplements the contact tracing investigations, as it highlights connections that were not identified through patient and contact interviews.

Our findings stress the importance of maximising the proportion of reported cases that get sequenced during EVD outbreaks. In this study, only a small fraction of the cases identified from the contact tracing investigations were matched to a viral sequence (20 per cent). Even in the transmission chains selected for the analysis, the majority of cases were not sequenced (between 38 and 54 per cent of cases per chain were sequenced). Increasing the proportion of sequenced cases would increase the information brought by inference methods and facilitate the comparison between contact tracing investigations and genomic data. The results we present show that the additional information brought by sequence data during EVD outbreaks is crucial to narrow down the pool of potential transmitters for a given case and identify transmission dynamics missed by the contact tracing investigations. It is, however, important to note that genomic data must be available and interpretable by contact tracers in near-real time to be useful as a control tool in outbreak situations, where time is key. Maximising the proportion of sequenced cases could also help identify different modes of transmission. Currently, discrepancies between phylogenetic and epidemiological reconstruction of two sequenced cases could be attributed to unsequenced infectors (either among the cases or common ancestors). If the proportion of sequenced cases had been higher, such a difference could have been attributed to different modes of transmission that have not been considered during the contact tracing investigations.

Our results also indicate that using the timing of infections alone was not sufficient to gain insights into the transmission dynamics in this dataset, and we believe that more epidemiological variables would need to be integrated in the model to improve the accuracy of the transmission tree inference. For instance, integrating spatial information or age group stratification of the cases into the likelihood of connection among cases may help disentangle the different independent transmission chains that are co-circulating at a given time and identify imported cases with better accuracy (Robert et al. 2020). However, this would require adding prior information or estimating the movement among regions (to study the impact of the location of the cases) or the number of contacts between age groups in the country, information that can prove difficult to collect or infer. Therefore, this would be especially relevant if the outbreak was contained in a smaller, ‘enclosed’ environment (as in the case of outbreaks in schools or hospitals) or if precise contact or movement information is routinely collected through modern technologies (e.g. air traffic records, contact tracing applications or devices, and social media data).

Although the conclusions of this study may be generalisable to other EVD outbreaks, or to other pathogens, it is important to note the impact of various factors on the reconstruction. First, factors specific to the life cycle of the studied pathogen (here EBOV) can influence the performance of the reconstruction methods and the phylogenetic approach (Campbell et al. 2018b; Huber et al. 2022). For instance, the rate of evolution of the pathogen of interest needs to be sufficiently high to generate population diversity within short time frames. This background diversity needs to be large enough to allow the identification of genomes that are more closely related to each other than you would expect by chance, a criterion indicative of recent transmission among the sample cases. Reciprocally, the generation time of the pathogen of interest should be long enough to allow the acquisition of informative nucleotide substitutions. In the case of EBOV, the distributions of the incubation period and the serial interval were wide, resulting in multiple likely infectors to be reported over a large time period. A pathogen with shorter and narrower distributions may be more adapted to the epidemiological approach. Second, factors related to the settings and context may have favoured certain inference methods: for instance, a strictly epidemiological approach may have performed better if there had been fewer concurrent chains over a longer time span.

In this study, we took the decision to only include sequenced cases in the final dataset in order to ensure that the likelihood of connection between cases was comparable. Otherwise, connections among sequenced cases would automatically be penalised ($\Omega = 1$ if one of the two linked cases is unsequenced, $\Omega \gt 1$ otherwise), which could introduce biases. Working towards a better inclusion of sequenced and unsequenced cases is an area of improvement for future analyses and would be crucial to make sure that the reconstruction analyses incorporates as many reported cases as possible.

## Supplementary Material

vead007_Supp

## Data Availability

The epidemiological and sequence data cannot be shared publicly for ethical reason. To make this study as reproducible as possible, we generated simulated transmission chains and applied the same analysis plan. The code used to generate the simulated chains and all the figures presented in the paper are shared in the following Github repository: https://github.com/alxsrobert/evd-transmission-trees.
