# Correction to: Quantifying the value of viral genomics when inferring who infected whom in the 2014–16 Ebola virus outbreak in Guinea

**DOI:** 10.1093/ve/vead021

**Published:** 2023-03-27

This is a correction to: Alexis Robert, Joseph Tsui Lok Hei, Conall H Watson, Pierre-Stéphane Gsell, Yper Hall, Andrew Rambaut, Ira M Longini, Jr., Keïta Sakoba, Adam J Kucharski, Alhassane Touré, Sévérine Danmadji Nadlaou, Mamadou Saidou Barry, Thierno Oumar Fofana, Ibrahima Lansana Kaba, Lansana Sylla, Mohamed Lamine Diaby, Ousmane Soumah, Abdourahime Diallo, Amadou Niare, Abdourahamane Diallo, Rosalind M Eggo, Miles W Caroll, Ana Maria Henao-Restrepo, W John Edmunds, Stéphane Hué, Quantifying the value of viral genomics when inferring who infected whom in the 2014–16 Ebola virus outbreak in Guinea, *Virus Evolution*, Volume 9, Issue 1, 2023, vead007, https://doi.org/10.1093/ve/vead007

In the originally published version of this manuscript, the images for Figure 5 and Figure 6 were transposed: these are now correctly positioned above their respective legends. There was also an error in an element within section **Keywords**, which should read: ‘ebola virus (EBV)’ instead of: ‘ebola virus (RSV)’.

The publisher apologises for these errors which have been emended within the article.

