# Supplementary material for: Quantifying the value of viral genomics when inferring who infected whom in the 2014–16 Ebola virus outbreak in Guinea
Source: Virus Evol. 2023 Mar 7;9(1):vead007. doi: 10.1093/ve/vead007 (PMC10013732; doi:10.1093/ve/vead007)
Supplement: vead007_Supp [file vead007_supp.zip › Final_Supplementary_material.Robert.EBOV_Transmission_Chains_Reconstruction_AR_SH.docx]

**Supplementary information**

**
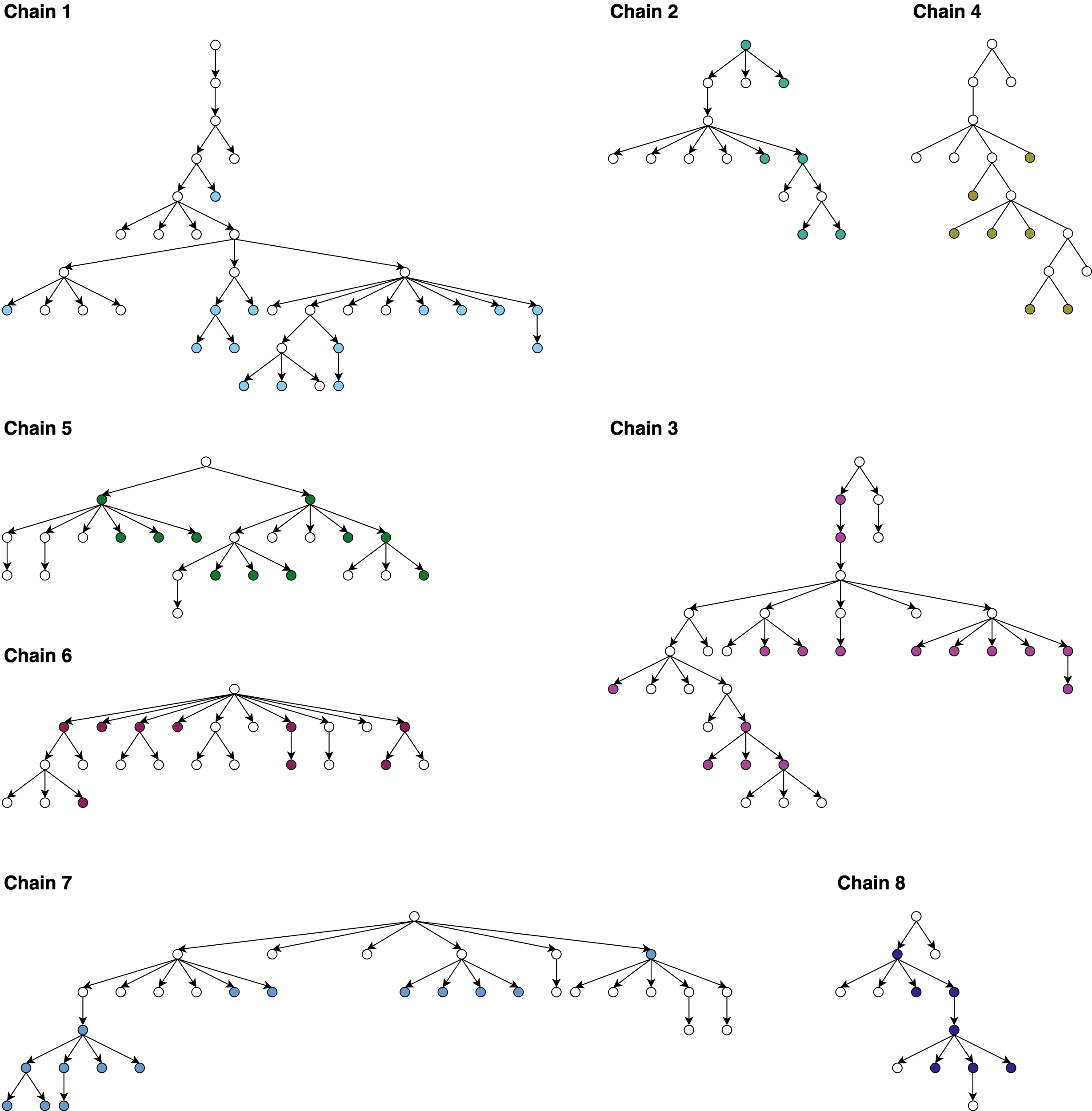
**

**Supplementary Figure 1.** Description of the 8 Ebola transmission chains determined by contact-tracing included in the analysis. Each node corresponds to a confirmed EBOV positive case and links between nodes correspond to contact between cases as determined by on-site-contact tracing. Coloured nodes correspond to cases with sequenced EBOV genomes.


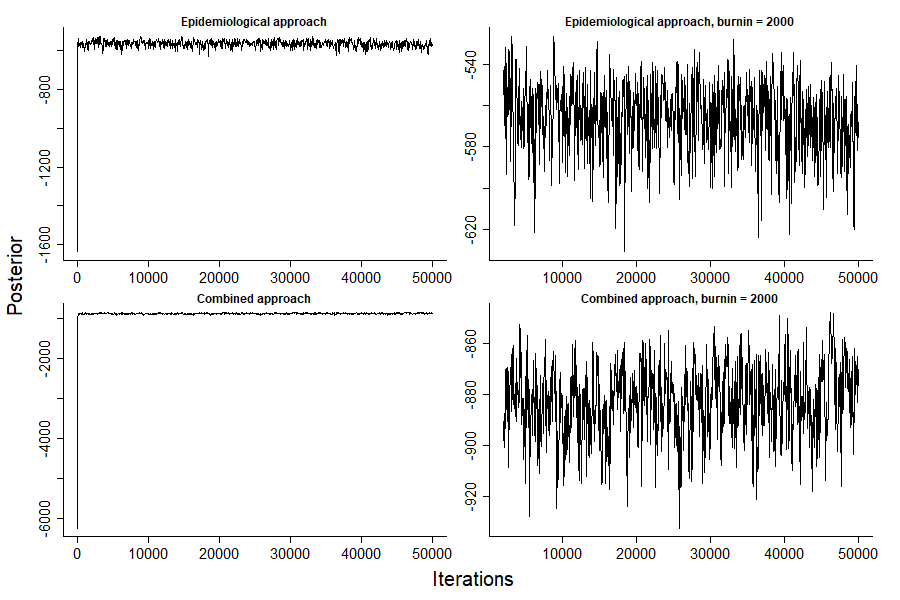


**Supplementary Figure 2.** Trace of the posterior value of the MCMC runs before and after removing the burnin section in the Epidemiological and combined approaches.


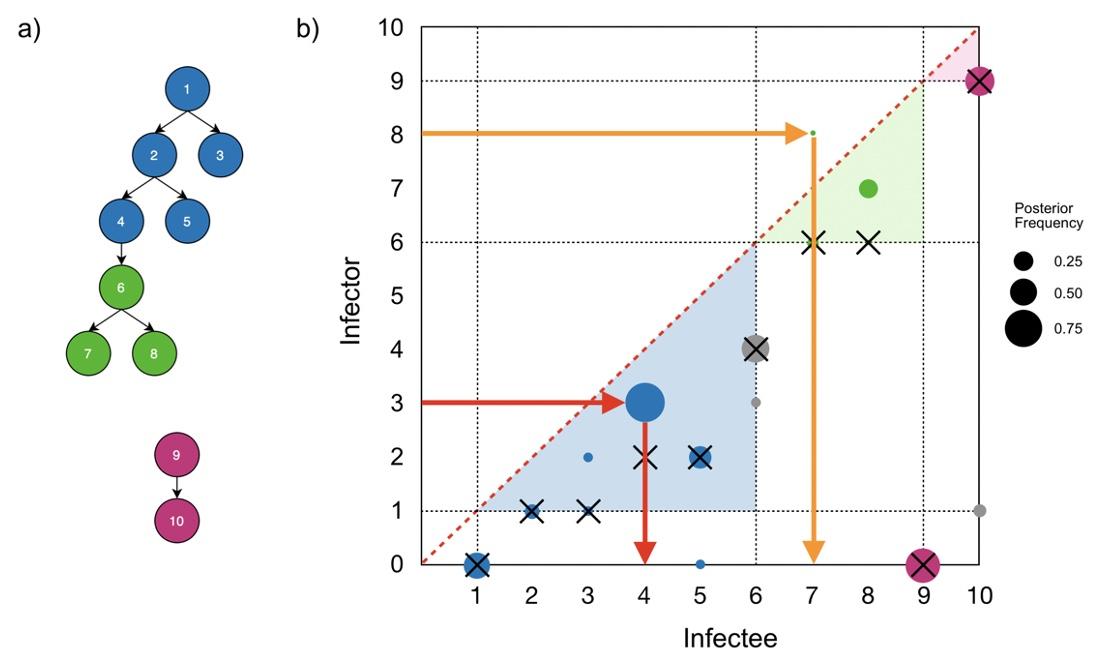


**Supplementary Figure 3**. Example Alpha-plot for a small sample dataset. a) Transmission tree reconstructed from a posterior sample. Each node is coloured coded by its corresponding chain of transmission as assigned by contact tracing (blue: chain 1, green: chain 2, purple: chain 3). In this particular posterior sample, chain 3 is not linked to either chain 1 or 2. b) Posterior distribution of ancestry assignments visualised in an alpha-plot with cases sorted by dates of symptom onset and chain number. The radius of each circle is proportional to the frequency of its corresponding transmission pair in the posterior distribution. Each circle is also colour coded by the chain of transmission to which the involved cases were assigned by contact tracing, e.g. a circle is coloured blue if both the infector and infectee were assigned to chain 1, and grey if the infector and infectee were not assigned to the same chain, indicating a cross-chain transmission. The crosses indicate the ancestry assignments in the posterior tree sample visualised in a). Having an infector labelled “0” (bottom row) indicates that the infectee is an imported case with an infector that is not observed in the dataset. The red dashed diagonal line indicates transmission pairs that are prohibited since a case cannot infect itself. The shaded triangles indicate regions where most transmission pairs are intuitively expected to be located given that cases are sorted in chronological order of symptom onset within each chain. E.g. the red solid arrows indicate a transmission from case 3 to case 4, with case 3 having symptom onset that predated case 4; the orange solid arrows indicate a transmission from case 8 to case 7 which is unexpected given that case 7 started developing symptoms earlier or on the same day as case 8 did (and so it lies outside the shaded region).

**
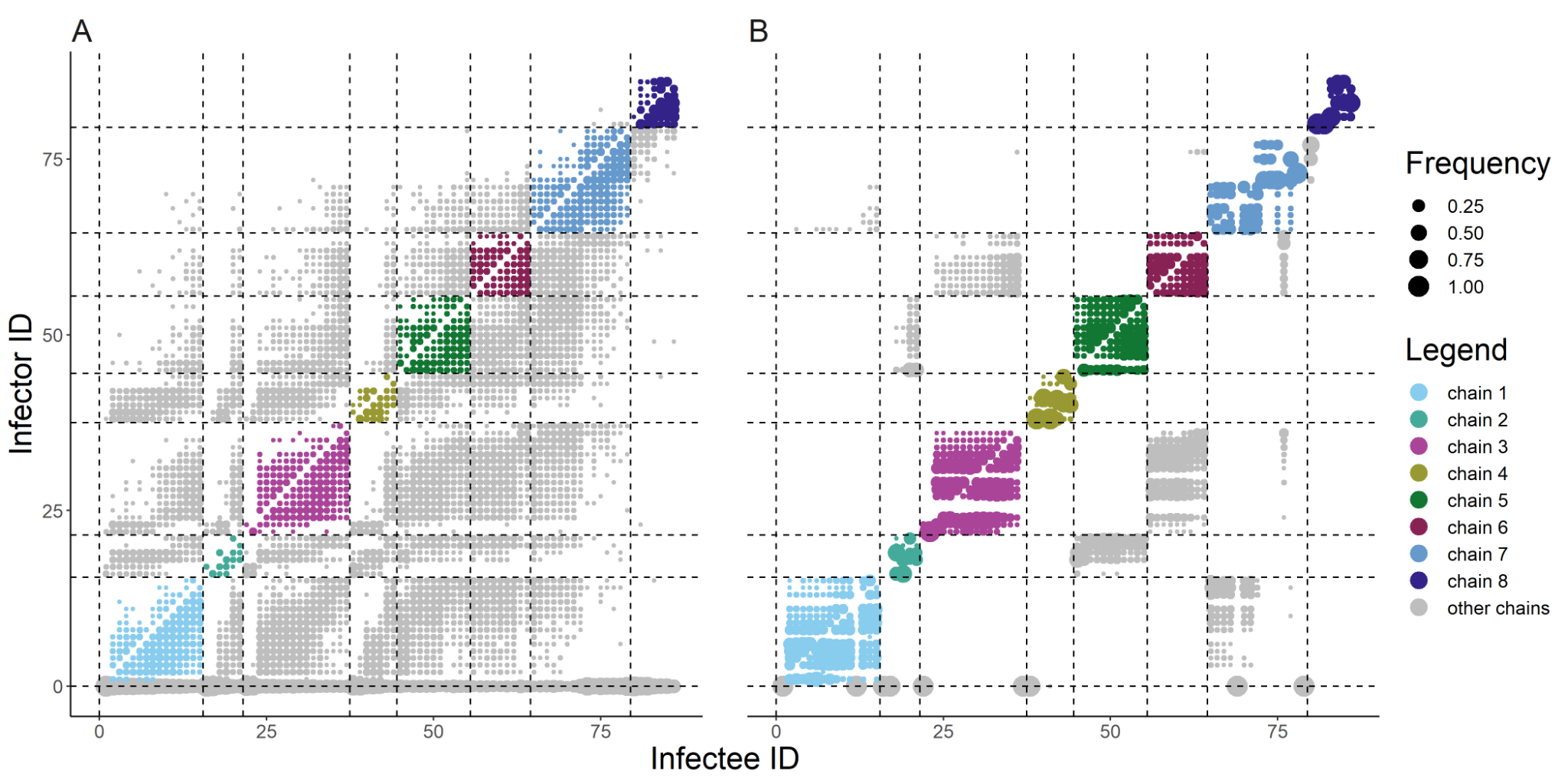
Supplementary Figure 4.** Impact of increasing the number of importations in the inferred trees: Alpha plot representing the posterior distribution of ancestry assignments obtained using the dates of symptom onset as the only data source (Panel A), and the dates of symptom onset and the genetic sequences (Panel B). The radius of each circle indicates the frequency of a given transmission pair in the posterior distribution (i.e. posterior frequency). An imported case is assigned an infector of index 0. Circles representing within-chain transmission pairs are colour-coded by chain of transmission. Circles representing cross-chain transmission pairs are coloured grey.

**
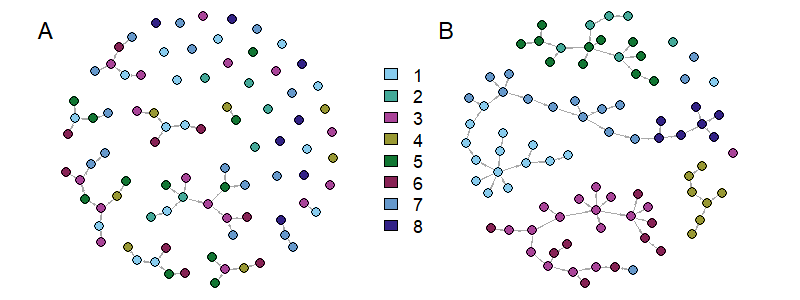
**

**Supplementary Figure 5.** Impact of increasing the number of importations in the inferred trees: Consensus transmission trees inferred in each approach. Each circle represents a case, and each case is connected to its most likely infector in the posterior distribution of ancestry assignments (the direction of the arrow indicates the infector-infectee relationship). Circles are colour coded according to their transmission chain in the contact tracing investigations (see **Figure 1**). (A) Consensus transmission tree obtained with the Wallinga Teunis algorithm; (B) Consensus transmission tree obtained with the combined approach.

**
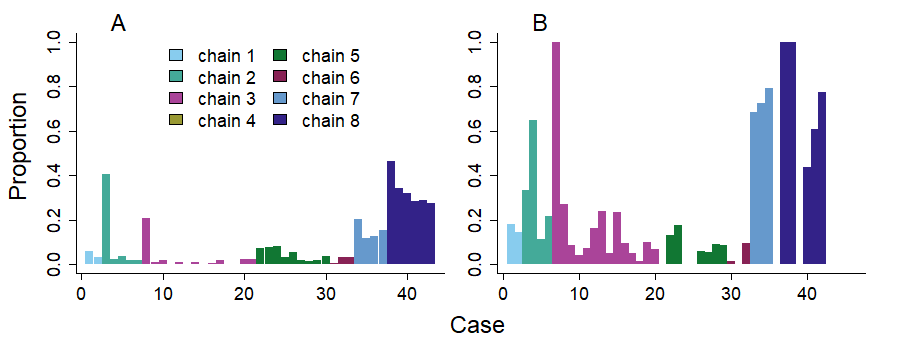
**

**Supplementary Figure 6.** Impact of increasing the number of importations in the inferred trees: Proportion of sampled trees where the cases are linked to their contact-tracing infector, in the epidemiological approach (Panel A), and the combined approach (Panel B).
